# ‘In the Midst of a Thunderstorm’: Young People's Experiences of Physical Restraint in Inpatient Mental Health Services in the UK

**DOI:** 10.1111/inm.70209

**Published:** 2026-01-09

**Authors:** Simon Nielson, Lucy Bray, Bernie Carter, Joann Kiernan

**Affiliations:** ^1^ School of Allied Health Professions and Nursing University of Liverpool Liverpool UK; ^2^ Faculty of Health and Social Care Edge Hill University Ormskirk UK; ^3^ Alder Hey Hospital Liverpool UK

**Keywords:** child and adolescent inpatient services, mental health services, physical restraint, restrictive interventions, young people

## Abstract

Within inpatient mental health services, young people who express distress through behaviours may be physically restrained. Little is known about their experiences or perspectives of this restraint. This qualitative Interpretative phenomenological analysis (IPA) study explored young people's experiences of physical restraint. Young people were recruited from three inpatient mental health units in England. Individual, face‐to‐face augmented, audio‐recorded semi‐structured interviews were undertaken. IPA data analysis facilitated the development of subordinate and superordinate themes. The study design was informed by public consultation with young people and their families. Eight young people (five boys, three girls, aged 10–13 years) shared their experiences of physical restraint. The findings are presented within the trajectory of a thunderstorm within three themes: The Gathering (‘pre‐escalation’), The Thunderstorm (the restraint), and The Aftermath (‘debriefing and making sense’). Young people talked about how feelings of being restrained could start long before any physical touch and could continue long after the physical element of the restraint had ended. They described emotional, traumatic and confusing experiences of restraint and often being left with emotional ‘debris’ for a long time after the incident. They also described situations where physical restraint was used instead of an investment in de‐escalation strategies. The current understanding of the trajectory of physical restraint for children and young people within mental health units needs to be adapted to recognise the extended gathering and aftermath stages associated with this intervention.

## 1 Introduction

Within the UK, inpatient children and young people's mental health services (CYPMHS) offer 24‐h care for children and young people aged under eighteen who have difficulties with their mental health or wellbeing (Health Education England 2024). Multi‐disciplinary teams within CYPMHS care for children and young people with a wide range of emotional, mental health and psychiatric issues (Association of Child and Adolescent Mental Health 2021). Restraint is often used within these services (Moell et al. 2024) in response to children and young people who express their distress through behaviours that can harm themselves or cause harm to others. In the UK, the Mental Health Units (Use of Force) Act 2018 defines physical restraint as the use of physical contact that is intended to prevent, restrict or subdue movement of any part of the patient's body (Department of Health and Social Care 2021). There is a drive to examine and reduce the use of restrictive interventions including physical restraint (Baker et al. 2021). To date this has focussed largely on the use of restrictive practices and restraint within adult settings (Bennetts et al. 2024) and little is known about the experiences of children and young people who have been physically restrained within healthcare settings, particularly mental health services.

## 2 Background

Physical restraint is a restrictive intervention that may be used in inpatient mental health settings to manage immediate risks of harm (Care Quality Commission (CQC) 2017; Department of Health (DH) 2015). National guidance and professional standards emphasise that restraint should only be employed as a last‐resort measure, once other non‐coercive strategies such as de‐escalation, environmental adjustments, or individualised behaviour support have been attempted or judged ineffective (CQC 2017; Department of Health and Social Care (DHSC) 2019). This approach reflects both ethical and regulatory imperatives to prioritise the least restrictive practice, ensuring that any use of restraint is proportionate, justified, and minimises potential physical and psychological harm. Contemporary approaches identify the need to reduce the use of restrictive interventions, including the use of physical restraint, as a key priority (DHSC 2019; Duxbury and Jones 2017), with providers tasked with adopting the least restrictive strategies (CQC 2017; DHSC 2019; Snipe and Searby 2023).

Strategies implemented to reduce the use of restrictive interventions, including restraint, include de‐escalation to prevent crises developing (Goodman et al. 2020) as well as improving the sensory environment by adapting the physical and sensory characteristics of the ward so that it becomes less overstimulating and more regulating for children who may be sensitive to noise, lighting, movement or crowding (Williams et al. 2023; Clement et al. 2022). Carefully planned and implemented post‐incident support/debriefs involving children or young people can also help reduce both the frequency and duration of subsequent physical restraint experiences (Eblin 2019). Approaches such as Positive Behaviour Support aim to avoid using physical restraint through understanding the function behind behaviour of the young person that is deemed challenging (Gore et al. 2013). Models used to frame a restraint trajectory, such as Kaplan and Wheeler's (1983) Assault Cycle consisting of five stages (trigger, escalation, crisis, recovery and post‐crisis phases) and the Safewards model (Bowers 2014) are frequently included within paediatric restraint training, despite being underpinned by evidence from adult populations.

Reducing restrictive practice is important. Physical restraint has been associated with emotional distress with adults with intellectual disabilities (Fish and Culshaw 2005; Hawkins et al. 2005) and adult mental health patients (Douglas et al. 2022), who have described experiencing feelings of sadness, distress, anxiety, anger and fear after being physically restrained (Laugharne et al. 2012; Merineau‐Cote and Morin 2013). Several studies have reported staff experiences of implementing physical restraint in healthcare settings (Kodua et al. 2023; Meehan et al. 2022; Krieger et al. 2021; Siegrist‐Dreier et al. 2022). Whilst there is some evidence to suggest that children and adults can have some shared experiences of physical restraint in healthcare (Hollins 2017; Scheuermann et al. 2016), there remains a gap in the literature specifically related to children's experiences of being physically restrained in inpatient mental health services.

Traumatic experiences during childhood, such as being physically restrained, can have adverse, far‐reaching effects on a young person's health and well‐being (Choi et al. 2019). When a child is physically restrained both they and the healthcare staff are at risk of physical harm (Nunno et al. 2022). However, solely focusing on physical harm can result in psychological harm being neglected (Bray et al. 2015).

Quantitative research has examined patterns of physical restraint among young people in US mental health residential treatment centres (Braun et al. 2020; Foster et al. 2023). In the UK, qualitative work has explored the use of physical restraint with inpatient adolescents (Kodua and Eboh 2023), although this has been limited to staff perspectives. Research in educational settings has also highlighted predominantly negative experiences among restrained pupils (Hodgekiss and Harding 2023). Systematic reviews conducted in the UK (Nielson et al. 2021) and Norway (Slaatto et al. 2021), reviewing literature from Israel, USA, Australia, Norway, Canada, Belgium, New Zealand and Finland, similarly conclude that further research is needed to understand children and young people's lived experiences of physical restraint, noting that they are not consistently asked to describe these experiences. Without a clearer account of how physical restraint is experienced by children themselves, care providers cannot fully appreciate the nature or extent of the harms associated with restrictive physical interventions. This gap underscores the necessity and contribution of the present study.

## 3 Research Question

This study aimed to answer the research question: what are the experiences of young people of being physically restrained during an admission to inpatient CAMHS and what are the effects (if any) that this physical restraint may have had upon young people's reported physical, social and emotional wellbeing?

### 3.1 Public Engagement

The public engagement which informed the design of this study is reported according to the GRIPP2 Short form (Staniszewska et al. 2017) (see Table 1). A patient and public engagement event was held by invitation from a list of volunteers generated through contacts of the primary investigator. As this phase of the study did not involve data collection to answer the research question, no formal consent or assent process was required. However, to ensure ethical transparency, all children and their parents or carers were provided with clear information about the study and what participation in the PPIE consultation would involve. The event was facilitated in a private area of a tea shop in the Northwest of England with nine young people (10–13 years) with and without first‐hand inpatient mental health experience and eight family members (six parents and two aunts). Family members engaged collaboratively alongside the young people using poster pages across several workshop stations. The language and terminology used emerged as imperative. Adults suggested avoidance of words such as ‘restraint’. However, young people with and without direct experience of inpatient mental health services and physical restraint unanimously disagreed, noting ‘why bother calling it “holding” when everyone knows it's “restraint”*’*. This discussion guided the terminology used within interview questions and participant information sheets. The researcher primarily used the term ‘physical restraint’ in the study, as this reflects the formal terminology used within policy, practice and the wider literature, and maintains clarity about the gravity of the practice under discussion. However, some children were more familiar with, or more comfortable using, the term holding. In these instances, the researcher adopted the child's language to support rapport, ensure that questions were meaningful to them, and avoid imposing terminology that might feel unfamiliar or overly formal.

**TABLE 1:** Gripp2 short form criteria.

Section and topic	Item
1: Aim Report the aim of PPI in the project.	To ensure meaningful engagement of young people and families in shaping study design (recruitment approaches, data collection methods) and materials (study information and activities) were accessible and engaging (particularly around language) and of minimum burden to those taking part for a sensitive topic (physical restraint)
2: Methods Provide a clear description of the methods used for PPI in the project.	A child‐ and family‐centred consultation event was held with young people (*n* = 9, aged 10–13) and family members (parents/family members *n* = 8). Conducted in a private café space with refreshments, drawing materials, and a flexible, obligation‐free format. Young people first engaged alone, then were joined by family members. A debrief session was facilitated at the end of the event to provide any additional support or clarification where needed
3: Study results Outcomes—Report the results of PPI in the project, including both positive and negative outcomes	Young people and families influenced language preferences, terminology, and interview style. Clear preference for the term ‘restraint’ over ‘holding’ was established. Insights shaped a semi‐structured interview schedule (10 broad questions). PPI also guided refinement of participant information sheets (simplified content, use of cartoons, improved child‐friendly language and fonts)
4: Discussion and conclusions Outcomes—Comment on the extent to which PPI influenced the study overall. Describe positive and negative outcomes	PPI enabled identification of child‐preferred terminology and sensitive approaches to interviewing, which may otherwise have been overlooked. Findings confirmed that young people with and without lived experience could provide valuable insights into the study design. Meaningful participation improved social value, design, and acceptability of the study
5: Reflections/critical perspective Comment critically on the study, reflecting on the things that went well and those that did not, so others can learn from this experience	Early, child‐centred consultation enriched the research design and materials. PPI strengthened ethical robustness, with the Health Research Authority recognising its contribution to minimising distress and improving risk–benefit balance. Despite some logistical barriers accessing this particularly vulnerable group, children's direct voices through PPI were essential in ensuring a respectful and appropriate methodology for this study

## 4 Method

### 4.1 Epistemological Position

This study adopts a constructivist epistemological position, which assumes that individuals actively construct meaning from their experiences within specific social and relational contexts. A qualitative approach, using Interpretative Phenomenological Analysis (IPA) (Smith et al. 2009), was selected as the methodology because it facilitates an in‐depth exploration of participants' lived experiences, emphasising the subjective and interpretive nature of meaning‐making. This enabled exploration of the lived experiences of young people through in‐depth semi‐structured face‐to‐face audio‐recorded one‐to‐one interviews. This approach is particularly appropriate for exploring complex, emotionally charged experiences such as physical restraint, where understanding the nuances of individual meaning is central to the study's aims.

### 4.2 Ethics Approval

The study received approval from the author's institution, Edge Hill University (the sponsor) (FOHSC 210; 20/06/18) and the Health Research Authority (IRAS 247096; 15/10/18). The Consolidated Criteria for Reporting Qualitative Research (COREQ) (Tong et al. 2007) checklist has been used to comprehensively and transparently report the study's methodology and findings (File S1).

### 4.3 Recruitment and Sample

Children and young people were recruited from three inpatient mental health wards, located across NHS hospital trusts in England. Inclusion criteria were young people aged 10–14 years, who had experience of physical restraint as an in‐patient on an inpatient mental health unit and with an imminent discharge date planned. Exclusion criteria were anyone for whom participation was deemed therapeutically contra‐indicated (as identified by the multi‐disciplinary team), any young person with current or potential safeguarding concerns, and those who had been involved in an incident connected to the service which was still under investigation.

Before a young person was approached to participate, written parental consent was required. Once this was obtained, the young person was given an information sheet, the opportunity to ask questions, and at least twenty‐four hours to consider participation. Great care was taken in terms of explaining their choices (declining, participating, withdrawing); if they wanted to participate, their written assent was gained. It was made clear to the young people that non‐participation would not be an influence on the quality of care they received during their admission.

### 4.4 Data Collection

Interviews were designed to facilitate rapport and trust‐building, and young people were eased into the topic of interest through drawing activities, which acted as a communication ‘bridge’ (Søndergaard and Reventlow 2019). Questions were open‐ended (File S2) and augmented by an optional creative activity (creating a timeline); this approach aimed to help children provide a detailed temporal description about their restraint experience (Zhang et al. 2019). The inclusion of flexible drawing activities aimed to enhance children's individual strengths and abilities. Fidget and construction toys were made available during the interview to support children who may have chosen to use these for regulation (Ciuffo et al. 2023). Closing questions were designed to provide clarification of the meaning behind experiences discussed whilst reorienting the young person away from potentially traumatic memorable events. Upon concluding interviews, children were reminded that they could approach staff if they experienced negative thoughts arising from their participation. Staff were made aware that participants might wish to seek additional support afterwards. Participant information sheets contained information about relevant trust‐specific support services, including crisis contact details and community resources.

Interviews were held in a quiet room on the mental health unit and the young person could choose to have their parent present or not. Interviews were audio‐recorded and were conducted by the lead author (male, white, mental health nurse not known to the young people or families).

### 4.5 Data Analysis

Adhering to the robust ideographic structure of Interpretative Phenomenological Analysis (Smith and Fieldsend 2021), analysis was undertaken on each transcript individually by the lead author (SN). At each stage of analysis, the developing analysis was independently checked and discussed with other team members (SN, LB, BC, JK). Initial line‐by‐line analysis resulted in more than 80 codes, which were grounded in the direct words of the children such as ‘they grabbed me’. Analysis progressed to clustering codes and then to themes, whilst retaining authenticity through using the children's words such as ‘you've got no control… you're being forced down’. As analysis progressed, a poignant quote from a child ‘It feels like a whirlwind in my mind’ led to exploration of the experience of being physically restrained being akin to being caught up in a thunderstorm. This quote supported the development of clustered, subordinate and superordinate themes.

### 4.6 Reflexivity and Researcher Positionality

The researcher's background as a mental health professional and academic with experience in child and adolescent inpatient services shaped both the design of this study and the interpretation of the data. Awareness of this positionality was central throughout the research process, prompting careful reflection on how prior knowledge, values, and assumptions could influence interactions with participants and analytic decisions. The researcher maintained a reflective journal throughout data collection and analysis to document thoughts, emotional responses, and emerging interpretations, providing a mechanism to critically examine potential biases and preconceptions. Additionally, the researcher engaged in ongoing supervision and discussion with academic mentors to challenge interpretive assumptions and ensure that participants' voices remained central to the analysis.

## 5 Results

**FIGURE 1:**
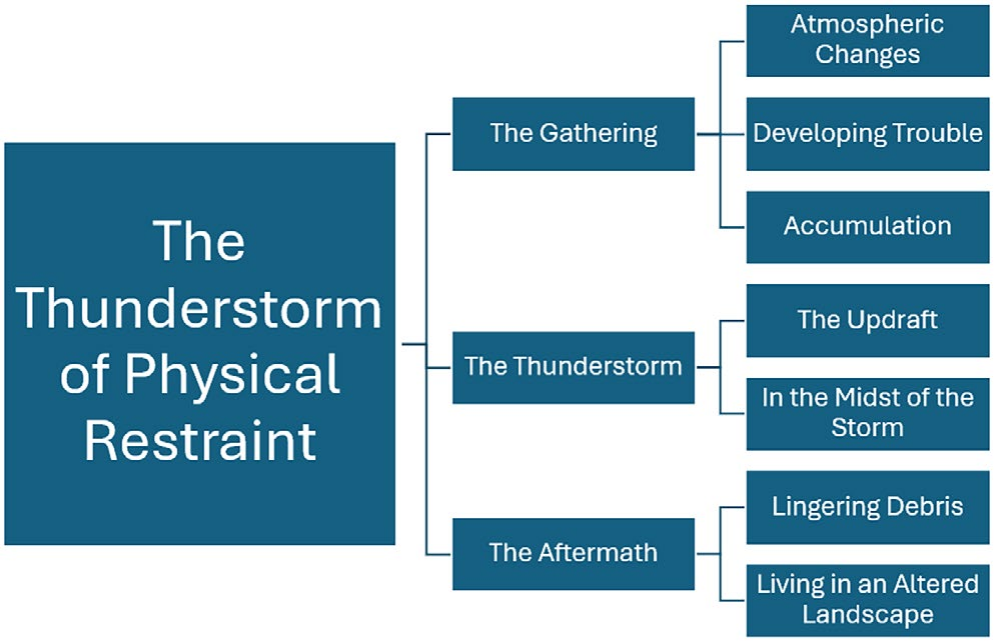
Overview of themes.

Eight young people participated in the study (5 boys and 3 girls, 10–13 years old); detailed demographics such as diagnoses are not reported to ensure anonymity. The findings are presented within the metaphor of the stages of a thunderstorm across three themes: The Gathering, The Thunderstorm, and The Aftermath. These themes reflect the trajectory of young people's experience of physical restraint (see Figure 1).

The young people chose pseudonyms and these are used within the findings: Battle Hound (boy), Nobody (girl), Mo Salah (boy), Clive (boy), Tik Tok (girl), Jay‐Z (boy), Knuckles (boy), and Lilleth (girl).

### 5.1 Theme 1; the Gathering

In most accounts, the young people described feeling a sense of trouble developing before there had been any actual physical contact. The Gathering involved notable atmospheric change, the development of trouble, and an accumulation of energy and a sense of unpredictability within the environment.

#### 5.1.1 Atmospheric Changes: ‘…You Can Feel It Ages Before It's Going to Happen…’

Clive, Lilleth, Knuckles, and Tik Tok picked up on very early signs of possible restraint from both actions by staff and from internal feelings:

I mean the night before they told me it was all getting… bad… they said they were… like it was…going to get worse if I ca… if I carried on with it…. (Clive)

Some young people described a palpable rise in their anticipation or expectation in the moments prior to being physically restrained where they felt that ‘something’ was going to happen. Tik Tok provided insight into how staff attempts to ‘discretely’ position themselves in preparation for the implementation of a physical restraint, were obvious to her:

…the staff were all like standing up on this side of the room like putting their stuff away and getting ready and nodding to each other like… like losers … they think I don't know what's about to happen … it's not a secret … it's obvious when it will happen…. (Tik Tok)

#### 5.1.2 Developing Trouble: ‘…You Have to Do All the Things They Say…’

Young people described an internal, individual sense of foreboding associated with a feeling of being threatened. Lilleth associated being physically restrained with *‘ex..treme’* ultimatums. Tik Tok, Clive and Lilleth revealed how the potential outcome of what was developing was unwelcome, negative and rooted within their previous experiences of physical restraint:

…the more you know about getting held and restrained the … more anxious you can get and like worried and stuff…. (Lilleth)

Knuckles explained restraint as the outcome of his choices to follow rules, explaining ‘…you have to do all the things they say…’.

#### 5.1.3 Accumulation: ‘…It's Too Late Now…’

The young people talked of accumulating feelings, thoughts and emotions (sadness, frustration, vulnerability, stress, anger and worry) in the moments prior to being physically restrained:

…but… they don't understand… they don’… they… I sleep with ‘im because I'm scared … being in this hos… this place… makes me more scared…. (Battle Hound)

Recognition of an accumulating, deteriorating situation helped some young people to prepare with a sense of inevitability about imminent physical restraint:

…they'll be like ‘sorry… you'll have to calm down’ and I'll be like ‘forget it… it's too late now… where were you before all this started?’ and it's like ‘well this is going to happen again isn't it?’…. (Tik Tok)

However, some young people, like Nobody, described becoming oppositional as the situation deteriorated

…they were being annoying so I was being annoying back… they were stopping me… so… I was stopping them…. (Nobody)

### 5.2 Theme 2; the Thunderstorm

The Thunderstorm relates to the transitory moments immediately prior to (the Updraft) and then at the point of being in the physical restraint (In the Midst of the Storm).

#### 5.2.1 The Updraft: ‘…It Feels Like a Whirlwind in My Mind…’

The moments immediately before physical restraint were characterised by young people experiencing an elevated sense of confusion, unpredictability, perceived loss of choices and awareness of a rapidly deteriorating situation. They talked about feeling unable to follow the restraint plan being presented to them, as Mo Salah explained ‘I don't think your plans are working…’. Tik Tok recalled how her plan was at odds with the staff plan.

…I just wanted to… see my mum, but… they were like ‘if you want to see your mum then we can help you…’ But I wanted to see her… and I couldn't… I could just stay where I was then go back to my room later but they were like ‘if you want to see your mum we can bring her to you when we can see you're calm’…. (Tik Tok)

They all reflected on a sense of chaos and confusion as they got caught in the updraft of a physical restraint:

I couldn't see their eyes so basically I didn't really know basically… er… like what was going to happen…. (Knuckles)

Tik Tok talked candidly about the impact upon her thoughts when she had felt that the staff had not helped her early enough:

…it feels like a whirlwind in my mind it… your head starts to spin off your shoulders and… then the nurses mess it all up and make it messy … I couldn't think of anything worse … my head feels like it's going to fall off my shoulders … it's not good. (Tik Tok)

Some young people described ultimatums and de‐escalation attempts by the staff as a ‘waste of time’ (Nobody) as staff did not understand or respond to their needs—‘it can be difficult to hear what they're saying sometimes…’ (Clive). Ultimatums resulted in lack of choice. Battle Hound explained that staff using a countdown to implementing restraint gave him no real choice:

…and then [name of HCP] is …standin’ away over here kinda… tellin’ everyone… what to do… and tellin’… me that I've got… a choice… but… I haven't got a choice… have I?… because when they start counting… that's their choice… not mine…. (Battle Hound)

#### 5.2.2 In the Midst of the Storm: ‘…Everyone Could See the Mess I Was in…’

When the young people talked about the physical element of being restrained, their communication was highly animated and energetic and characterised by pauses, mispronunciations, distorted timelines, repetition and corrections. They all talked about a perceived loss of power and control through being physically restrained.

…it felt like I was falling down you know like in between them like being squashed down… like being forced down… it just felt like… pressure downwards…. (Tik Tok)

Lilleth nervously outlined her own experience of loss of power and control when she was being moved against her will.

… it was more like being dragged than being guided… two people came and then just dragged me off and… erm… then pinned me down…

Children's feelings of a loss of power, control and freedom were also associated with their feelings of a lack of identity.

…as soon as they go to grab you, you do what you can to get away… I just got caught by my shoulder when I tried to run away… and they were like ‘don't worry, don't worry we'll deal with it’ and I remember thinking ‘what are they talking about?… what are they dealing with?… Me? It's just… it's just stupid’…. (Clive)

Anger and fear were commonly associated with being physically restrained, frequently as a precursor, but also through being maintained or magnified as a result of restraint:

… angry… and kind of… not so much upset but I felt kind of… scared…. (Mo Salah)

Jay Z, Knuckles, Mo Salah, Battle Hound, Clive, Tik Tok and Lilleth all talked about being surrounded by noise whilst they were being physically restrained:

…I didn't really know what was going to happen… I'm seriously getting a lot of talking at me… I wish they helped me, you know, like, I wished they helped me… more…. (Knuckles)

Battle Hound and Clive highlighted the potential for embarrassment during physical restraint:

…it was embarrassing… because [name of a patient] was there and he's my friend and then I was getting… like sent to my room like a little kid…. (Battle Hound)

Clive, Tik Tok, Lilleth, Knuckles and Jay‐Z described reaching a personal tipping point, a sense of hopelessness, and giving up their fight with the staff who were physically restraining them.

…I remember just looking at the clock and hoping it would turn around quickly and I was saying to myself ‘please go around quickly I hate this… please go around’…. (Tik Tok)

Knuckles described feeling like the staff were not listening to him, and, in the end, resorted to pleading with his mother to intervene and stop the restraint:

…and I was telling [HCP] to stop it… I just want them to stop it … I asked my mum to please stop it…. (Knuckles)

### 5.3 Theme 3; the Aftermath

Being in the Aftermath for children was about making sense of Lingering Debris; re‐visiting and reflecting upon their physical restraint experiences and seeking clarification and understanding to prepare them for Living in an Altered ‘post‐restraint’ Landscape.

#### 5.3.1 Lingering Debris: ‘…I Survived… I Survived… I Got Through It…’

Clive, Nobody, Lilleth, Tik Tok, Battle Hound and Knuckles all appeared to be either still dealing with, or living with, the aftermath of physical restraint. Some were still sifting through the lingering debris of difficult thoughts, feelings and memories about their experiences:

…I remember thinking ‘why do people do that?’ Why is it not calming… it's doing my head in… I don't understand why they have to keep doing that and not something better for me… like find a more compassionate approach for me… I literally get up in the morning and I'm… like… ‘what is this place?’…. (Tik Tok)

All the children appeared to have tried to make sense of their experiences. All the children apart from Jay‐Z, were left with confusing unanswered questions, and five felt that being physically restrained had either not helped them or had made their time in the hospital worse. Lilleth's experience of being physically restrained had fundamentally changed the way she viewed the world:

…now I… associate sitting down [laughs] with being in pain like… if anything that's made it worse… because like… I've got… that link of fear… with… just doing normal stuff… it's like… everything kind of links to… something bad…

Clive reflected on how being physically restrained had set him back during his hospital admission:

…it was just not great and I'm just thinking… it just sets you back like… it's just like always them that…well… starts it like they probably just wanna be seen to have the control in front of the others…. (Clive)

Battle Hound, Jay‐Z, Clive, Tik Tok, Mo Salah and Lilleth referenced how they felt that there was value in talking things through after experiencing physical restraint.

…at this hospital we get to talk about it after… which is good because we can see what happened…. (Jay‐Z)

Clive, Knuckles, Mo Salah and Lilleth reflected about how they felt that they had coped well with difficulties that were being presented to them through their being physically restrained. Whereas Clive, Knuckles and Mo Salah described, in an indirect manner, their feelings of survival:

…and I just remember screaming and … it was just absolutely chaotic… but I… I… I survived… I survived I got through it…. (Lilleth)

Seeing other young people being restrained was normalised:

I get used to it… everyone gets used to it and it just becomes kind of nothing … it's just something that they do to us…. (Jay‐Z)

#### 5.3.2 Living in an Altered Landscape ‘…I'm Never Gonna Get This Time Back…’

Children and young people were clearly trying to make sense of their restraint experience by ‘clearing up’ and moving on. Time was an important factor for this. Clive reflected on how he would never get the time back that he had lost by being admitted to hospital

…it just sets you back like… I lost a lot of time in here like… I'm never gonna get this time back again am I?… like… it's been ok here but I will not miss it I mean like… it's safe but I haven't enjoyed it…. (Clive)

Clive, Tik Tok and Knuckles all talked about learning from their experiences and preparing to move on with their lives.

I guess the important thing is that I try not to get restrained any more … so I have to try and still think about when I'm going to leave…. (Tik Tok)

Knuckles emphasised the value of trying to listen to what staff were saying.

you just need to listen and… but it's hard… but you need to listen and erm like you can always try again for the ne… next time… if you listen to them…

## 6 Discussion

This is the first study to interview young people about their experiences of restraint during an admission to an inpatient mental health unit. To date, no models have been published to map the trajectory of how young people experience a restraint incident within healthcare settings. This study provides data to inform a model derived from the experiences of young people that challenges a reliance on the Assault Cycle model (Kaplan and Wheeler 1983), which is still used widely in the education and training of behaviour support for health professionals in the UK (Hallett and Dickens 2017).

**FIGURE 2:**
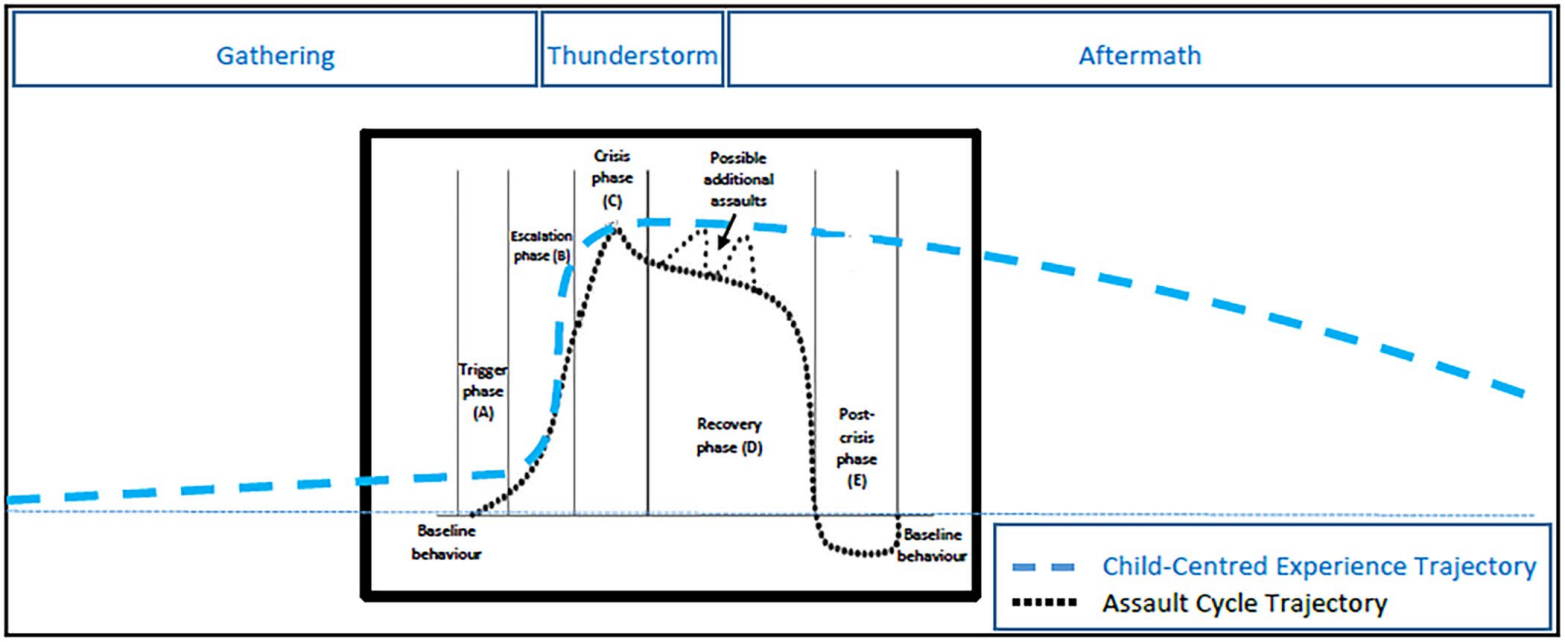
Trajectory of physical restraint as experienced by children and young people (mapped against the Assault Cycle).

The young people described difficult, emotional and confusing experiences of physical restraint. These descriptions led to the conceptualisation of a young person‐centred model of the trajectory of physical restraint that goes beyond the existing models. Centring on the concept of a thunderstorm, the model encompasses three stages: The Gathering (escalation); The Thunderstorm (restraint); and The Aftermath (recovery, post‐crisis). What is particularly notable with this model, when compared with other models such as the Assault Cycle (Kaplan and Wheeler 1983), is that the period of restraint (The Thunderstorm) is relatively short compared to the stages which come before and after. These stages are also important for the young people and run over an extended period (hours and days) of time (see Figure 2).

Within The Gathering, the young people sensed the build‐up of tension and anxiety well before physical restraint occurred, and they described how threats and ultimatums were sometimes used by staff to control and coerce. This conflicts with the advice that a culture of care and prevention should be the first‐line intervention to override disruptive situations within a therapeutic approach (Duxbury and Jones 2017). Maintaining trust, care and connection are important elements to mitigate against escalating distress (Price et al. 2024), and this ‘pre‐escalation’ can be thought of as the phase prior to the escalation of emotions and/or behaviours (Healy et al. 2020). Beyond the pre‐escalation stage, distress and behaviour that challenges can, in theory, be managed through de‐escalation strategies or interventions such as Positive Behaviour Support designed to avoid the use of restrictive practices through understanding the individual function of a person's behaviour (Gore et al. 2013).

The young people, however, identified that de‐escalation was problematic, avoidable and suggested that physical restraint could feel inevitable. Staff were not described as exploring de‐escalation through tailored and individualised communication, and collaboration grounded in connection and trust (Bell and Condren 2016; Conn et al. 2017; Price and Baker 2012). As identified in other research, gaps in staff knowledge and skills were evident from the young people's accounts. A deficiency in training equipping healthcare staff to adequately adopt de‐escalation and pre‐escalation preventative strategies has been identified, particularly in emergency departments (Stene et al. 2015). Bowers et al. (2004) suggest that a focus on containment, rather than a therapeutic approach, is part of the problem of aggression and violence in clinical settings. Staff should be able to focus on the prevention of the onset of aggression in an informed, structured and caring manner (Duxbury and Jones 2017). Embedding this approach could shift a service culture away from ‘response and reactivity’ (de‐escalation) towards one of ‘prevention, engagement and proactivity’ (pre‐escalation).

The young people described physical restraint (The Thunderstorm) as traumatic, confusing and making the situation worse. Most studies focus on the physical impact of restraint (Nielson et al. 2021) overlooking the psychological harm (Bray et al. 2015). Restraint could be framed as an adverse childhood experience (ACE) that can lead to future negative impacts on well‐being (Tzouvara et al. 2023). ACEs are recognised within healthcare as events causing chronic stress responses in the child's immediate environment (Kelly‐Irving et al. 2013). Young people's descriptions revealed how the impact of physical restraint can linger for a long time (The Aftermath) as they tried to make sense of what happened, often on their own without any structured support or discussion. The model shows how young people did not describe returning to a level of ‘baseline behaviour’ after a restraint episode as noted in previous models, but carried an accumulation of distress forwards with them. Although evidence suggests that debrief can help young people to make sense and re‐frame traumatic experiences (Steele and Kuban 2014), Larue et al. (2010) note that post‐incident debriefing tends to focus more on staff support than on a young person's traumatic experience.

Although this study did not include adult participants, the findings highlight distinctions that may be particularly relevant when comparing children's and adults' experiences of physical restraint. For children, relational and developmental factors—including the tone of staff communication, the use of age‐appropriate language, non‐verbal cues such as eye contact and body positioning, and the maintenance of trust and connection before, during, and after restraint—appear central to their experience. These aspects may be less emphasised in adult‐focused models, which often prioritise containment and risk management. Recognising these differences underscores the need for child‐centred approaches to restraint that attend to both emotional and developmental needs, with implications for training, policy, and clinical practice.

The title of this study draws on a participant's own metaphor to convey the emotional intensity of their experience; it does not signify any conceptual alignment with any established models. The imagery is used purely to honour the child's language and to support a phenomenological understanding of their lived experience, rather than to invoke or apply an established theoretical framework. Therefore, this research provides new insights and a new model to challenge understandings of the experience of being physically restrained in hospital, which are currently dominated by adult‐centred incident‐management models.

## 7 Strengths and Limitations

The robustness of the study was guided by Yardley's (2000) four key characteristics of ‘sensitivity to context’, ‘commitment to rigour’, ‘transparency and coherence’ and ‘impact and importance’. A strength lies in the way the study has centred on respecting young people and supporting their participation. This has been through consultation with children and young people in the design of the study, materials and language used. Other strengths include the use of augmented interviews to facilitate participants to share their experiences on their terms. The collection of accounts from young people from three inpatient units allows insights across different sites to be gained. Whilst the sample size was small (*n* = 8) this aligns with IPA methodology. The positionality of the lead researcher as a mental health nurse means the collection and analysis of data was through this lens. This was mitigated by robust checks with other team members who were not mental health nurses.

## 8 Relevance to Clinical Practice

To improve care, staff should receive appropriate training when caring for young people within inpatient mental health settings to understand and gain skills in using techniques to prevent the use of restraint beyond de‐escalation, to recognise the context of ‘pre‐escalation’ and the importance of care, connection and trust in the trajectory of restraint for young people. Training should also help staff understand the long‐term impact of being physically restrained on young people and what it means to provide meaningful ongoing post‐incident support.

## 9 Future Research and Implications

Future research should further examine the experiences of children and young people across a broader range of inpatient mental health settings, including adolescent units, to understand developmental differences in how restraint is perceived and experienced. Longitudinal studies could explore the longer‐term psychological, emotional, and relational impacts of physical restraint, as well as the effectiveness of post‐incident debriefing and therapeutic interventions in supporting recovery. Research could also investigate the implementation and impact of child‐centred staff training programmes that emphasise pre‐escalation strategies, relational care, and the minimisation of restrictive practices, evaluating how these approaches influence both staff behaviour and young people's experiences. Such work would provide an evidence base to inform policy, service design, and clinical practice, with the aim of reducing the use of restraint and mitigating potential harm.

## 10 Conclusion

Young people within inpatient mental health units described emotional, confusing, and sometimes traumatic experiences of being physically restrained. The feelings associated with restraint typically started long before any physical touch and continued long after the ‘physical’ element of the restraint had ended. Young people were dealing with ‘emotional debris’ and feared staff for a long time after the physical restraint incident. Findings highlight the need for the current understanding of the trajectory of physical restraint to be adapted to recognise the extended gathering ‘pre‐escalation‐to‐de‐escalation’ and aftermath ‘debriefing and making sense’ stages as experienced by young people.

## Author Contributions

Conceptualisation: S.N., J.K., L.B., B.C.; participant screening: S.N.; data collection: S.N.; data analysis: S.N., J.K., L.B., B.C.; completing the first draft: S.N.; completing final draft: S.N., J.K., L.B., B.C. All authors meet the prerequisite authorship criteria and support the publication of the study findings.

## Funding

The authors have nothing to report.

## Conflicts of Interest

The authors declare no conflicts of interest.

## Supporting information


**Data S1:** inm70209‐sup‐0001‐Supinfo.docx.

## Data Availability

Data available on request due to privacy/ethical restrictions.
