# Supplementary figures and images for: ‘In the Midst of a Thunderstorm’: Young People's Experiences of Physical Restraint in Inpatient Mental Health Services in the UK
Source: Int J Ment Health Nurs. 2026 Jan 9;35(1):e70209. doi: 10.1111/inm.70209 (PMC12784178; doi:10.1111/inm.70209)

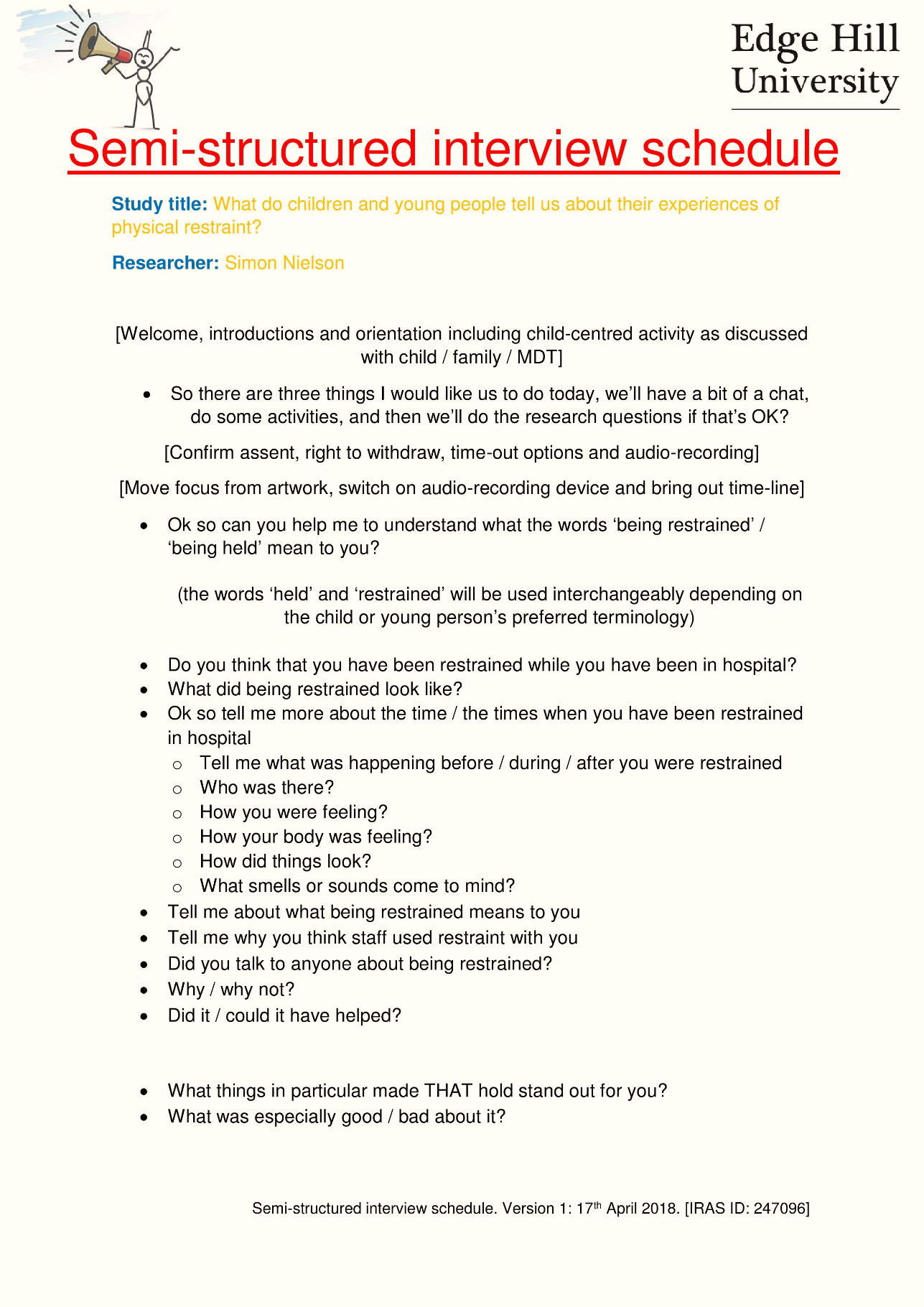


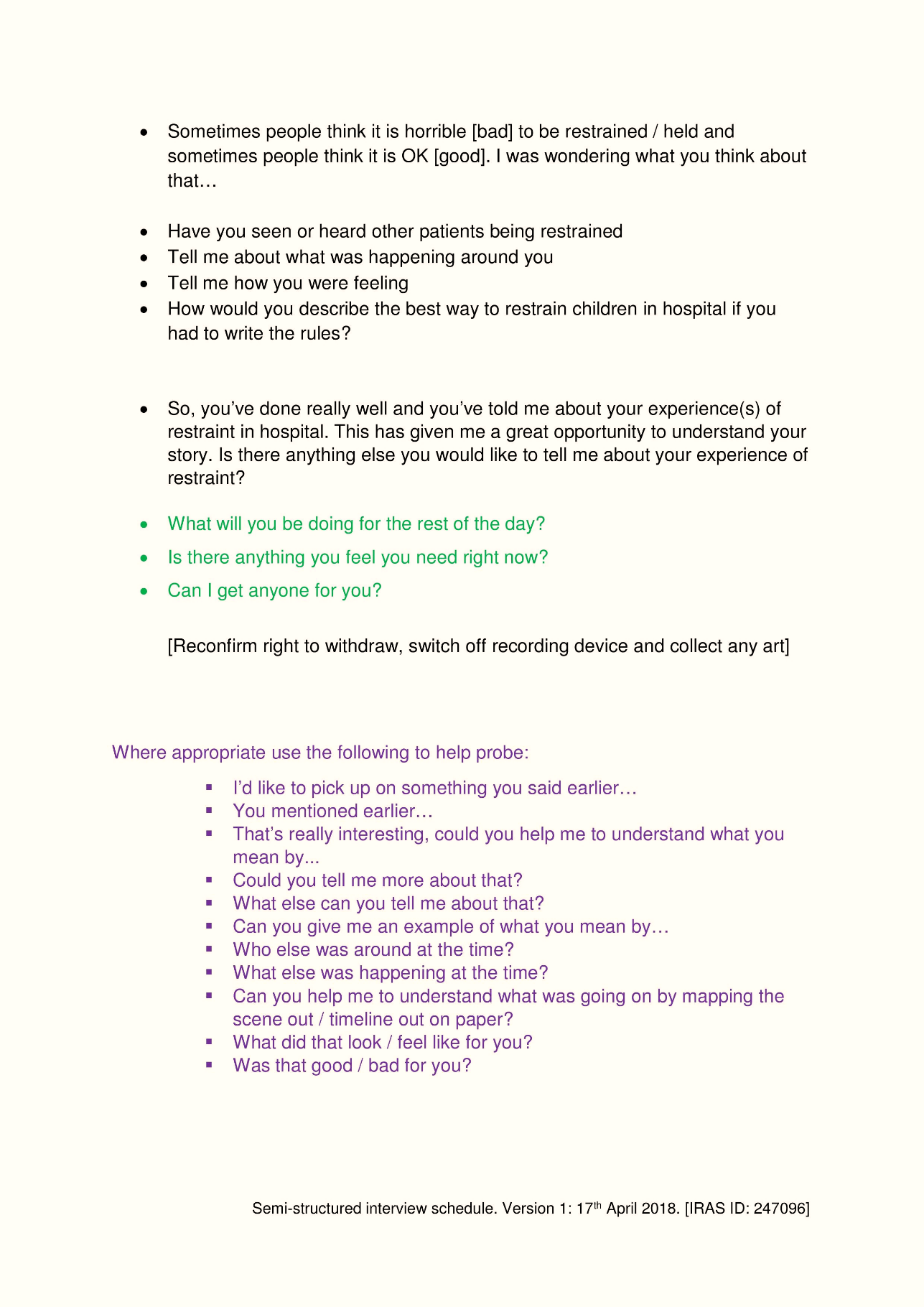

Supplement: Supplementary file 1 — Data S1: inm70209‐sup‐0001‐Supinfo.docx. [file INM-35-0-s001.docx]
